# Repeatability of Quantitative Autofluorescence Imaging in a Multicenter Study Involving Patients With Recessive Stargardt Disease 1

**DOI:** 10.1167/tvst.12.2.1

**Published:** 2023-02-01

**Authors:** Patty P. A. Dhooge, Philipp T. Möller, Nils Meland, Katarina Stingl, Camiel J. F. Boon, Andrew J. Lotery, Maurizio Battaglia Parodi, Philipp Herrmann, Wolfgang Klein, Mario G. Fsadni, Thomas H. Wheeler-Schilling, Frank G. Holz, Carel B. Hoyng, Steffen Schmitz-Valckenberg, for the Soraprazan Consortium

**Affiliations:** 1 Department of Ophthalmology, Radboud University Medical Center, Nijmegen, The Netherlands; 2 Donders Institute for Brain, Cognition and Behaviour, Nijmegen, The Netherlands; 3 Department of Ophthalmology, University of Bonn, Bonn, Germany; 4 GRADE Reading Center, Bonn, Germany; 5 Univeristy Eye Hospital, Center for Ophthalmology, University of Tuebingen, Tuebingen, Germany; 6 SMERUD Medical Research International AS, Thunes vei 2, Oslo, Norway; 7 Department of Ophthalmology, Leiden University Medical Center, Leiden, The Netherlands; 8 Department of Ophthalmology, Amsterdam University Medical Centers, Amsterdam, The Netherlands; 9 Faculty of Medicine, University of Southampton, Southampton, UK; 10 Department of Ophthalmology, Ospedale San Raffaele, Milano, Italy; 11 Center for Rare Diseases Bonn (ZSEB), University of Bonn, Bonn, Germany; 12 Katairo GmbH, Kusterdingen, Germany; 13 International Pharm-Med Ltd., Bramhall, UK; 14 Center for Ophthalmology and Institute for Ophthalmic Research, University of Tuebingen, Tuebingen, Germany; 15 John A. Moran Eye Center, University of Utah, Salt Lake City, UT, USA

**Keywords:** Stargardt, quantitative autofluorescence (qAF), autofluorescence(AF), repeatability, end point

## Abstract

**Purpose:**

This study assesses the repeatability of quantitative autofluorescence (qAF) in a multicenter setting and evaluates qAF as the end point for clinical trials in recessive Stargardt disease 1 (STGD1).

**Methods:**

A total of 102 patients with STGD1 underwent qAF imaging as part of the Stargardt Remofuscin Treatment Trial (STARTT; EudraCT No. 2018-001496-20). For 166 eyes, we obtained qAF imaging at 2 visits, with 2 recordings per visit. The qAF_8_ values were independently determined by the study site and a central reading center. Intra- and inter-visit reproducibility, as well as interobserver (study site versus reading center) reproducibility were obtained using intraclass correlation (ICC), one-sample *t*-test, and Bland-Altman coefficient of repeatability.

**Results:**

The qAF repeatability was ± 26.1% for intra-visit, ± 40.5% for inter-visit, and ± 20.2% for the interobserver reproducibility measures. Intra-visit repeatability was good to excellent for all sites (ICC of 0.88–0.96). Variability between visits was higher with an overall ICC of 0.76 (0.69–0.81). We observed no significant difference in qAF values across sites between visits (7.06 ± 93.33, *P* = 0.238).

**Conclusions:**

Real-life test-retest variability of qAF is higher in this set of data than previously reported in single center settings. With improved operator training and by selecting the better of two recordings for evaluation, qAF serves as a useful method for assessing changes in autofluorescence signal.

**Translational Relevance:**

The qAF can be adopted as a clinical trial end point, but steps to counterbalance variability should be considered.

## Introduction

Recessive Stargardt disease 1 (STGD1) is the most common inherited macular dystrophy that results in progressive vision loss.^1^^–^^3^ It is caused by mutations in the *ABCA4* gene, encoding the ATP-binding cassette transporter A4 (ABCA4), which plays an important role in the recycling of vitamin A. ABCA4 insufficiency leads to impaired removal of vitamin A derivatives from the photoreceptor disks and retinal pigment epithelium (RPE) lysosomes.^4^^,^^5^ Because of the impaired removal, metabolic byproducts of the visual cycle react to form A2E and related bisretinoid molecules. These molecules accumulate in the RPE as components of lipofuscin, subsequently resulting in degeneration of both RPE and corresponding photoreceptors.^6^^–^^8^ Until recently, this process was thought to be irreversible but different treatment options are currently being evaluated in clinical trials.^9^^,^^10^ However, sensitive end points to test STGD1 progression and the treatment effect of these newly therapeutic strategies have not been established yet.

Quantitative autofluorescence (qAF) acts as a direct biomarker of lipofuscin levels in the retina and might, therefore, be a suitable end point for clinical trials in STGD1.^11^^,^^12^ The retina emits an intrinsic autofluorescence signal upon excitation that originates principally from lipofuscin fluorophores and melanolipofuscin.^13^^,^^14^ Because of the increased formation of lipofuscin in STGD1, autofluorescence intensities are significantly higher compared to normal subjects with the same age.^15^^–^^17^ Recently, it has become possible to objectively quantify the autofluorescence signal in a standardized way by means of qAF. By including a constant reference fluorescence signal in the optical pathway of a confocal laser scanning ophthalmoscope, it has become possible to compensate for changes in laser power and detector gain.^18^^,^^19^ This also allows for the comparison of qAF images between different sites and different visits, which is essential for interventional longitudinal and multicenter trials.

Validation of a surrogate end point requires, among others, an investigation of repeatability.^20^ In this paper, we studied the repeatability performance of qAF in a multicenter setting in patients with STGD1 by using data from the Stargardt Remofuscin Treatment Trial (STARTT), EudraCT No. 2018-001496-20.^21^ Before the initiation of treatment, all patients satisfying the other enrollment criteria of this trial underwent qAF imaging at two visits, with two qAF imaging sets (referred to as “movies”) per visit. For each visit, the study site evaluated the qAF movie with the highest quality (according to subjective judgment). The reading center always graded both qAF movies. Within the STARTT, the reading center will select the movie with the highest qAF value to evaluate the efficacy of remofuscin. However, by using all the individual measurements, we were able to evaluate the intra-visit, inter-visit, and interobserver repeatability of qAF.

## Methods

This study used data from the STARTT with EudraCT No. 2018-001496-20. The study design with its inclusion and exclusion criteria has been described in detail previously.^21^ In brief, STARTT is an ongoing phase II, prospective, multicenter, randomized controlled trial to evaluate the safety and efficacy of oral remofuscin in patients with STGD1 (last patient and last visit expected in September 2022). From June 2019 to August 2020, patients were recruited from 6 European clinical study sites: Radboud University Medical Center Nijmegen, Nijmegen, the Netherlands; Leiden University Medical Center, Leiden, the Netherlands; University Eye Hospital Tübingen, Tübingen, Germany; Department of Ophthalmology, University of Bonn, Bonn, Germany; University of Southampton, Southampton, United Kingdom; and Ospedale San Raffaele, Milano, Italy. For this paper, sites have been anonymized using numbers #1 to #6 in a random order. The study was conducted according to the principles of the Declaration of Helsinki. For all sites, the study was approved by local institutional review boards. Written informed consent was obtained after explanation of the nature and possible consequences of the study. For all sites, the study protocol was approved by each of the institutional review boards.

Herein, we focus on key points relevant to qAF imaging analysis and included data from the screening visit (visit 1) and baseline visit (visit 2) of STARTT. Patients did not receive treatment between screening and baseline visits. For the screening visit, all study sites identified and recruited suitable patients with STGD1 from the site's database or newly presenting patients. Patients were asked to attend the baseline visit if they met all the previously described inclusion and exclusion criteria at the screening visit.^21^ Most importantly, to be included, the qAF_8_ value had to be ≥300 units in the study eye as determined at the screening visit and confirmed by the central reading center. The cutoff point of 300 units was determined as the lower limit because this value is above the physiological age-dependent increase of qAF8 levels and below the ceiling level of approximately 800 units previously measured in STGD1 eyes.^21^ The study protocol allowed an additional re-screening visit (visit 1B) to repeat and re-submit qAF images in case the reading center was not satisfied with the quality of the image set from the first visit. Visit 1b was only done when image quality was inadequate. Eyes that did not meet the 300-unit threshold with good quality qAF images, were not reimaged in another attempt to meet threshold values.

### Patient Selection

**Figure 1.**
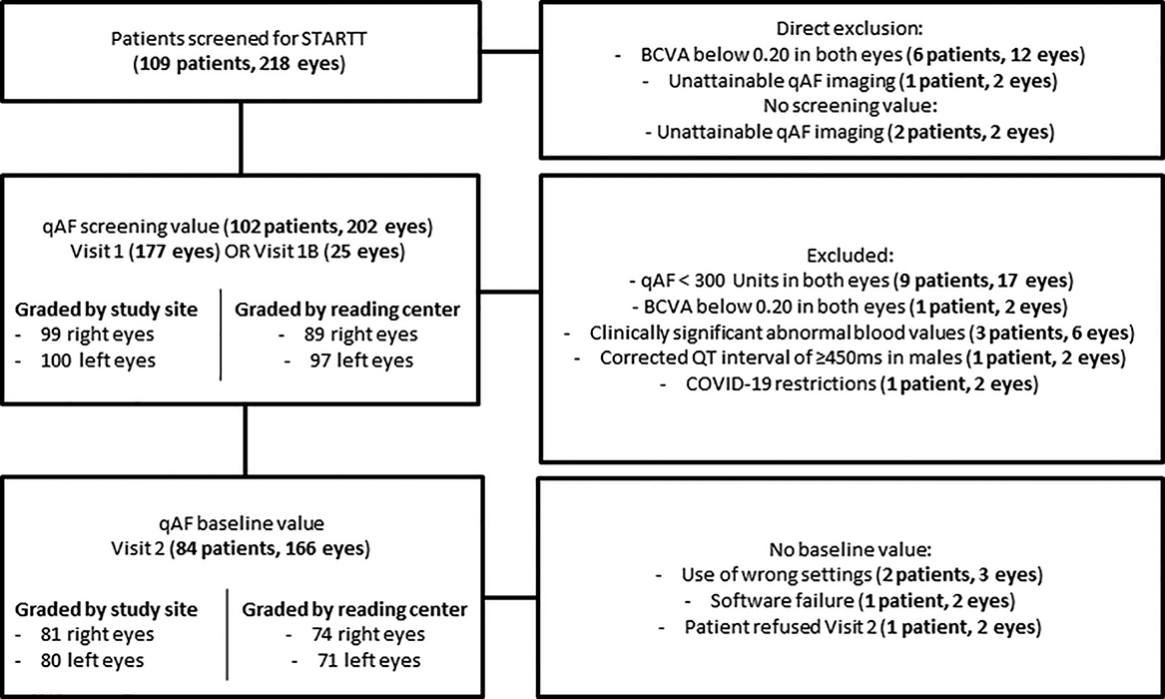
Included and excluded eyes. Quantitative autofluorescence (qAF) screening values were available for 202 eyes and baseline values were available for 166 eyes. Eyes were independently graded by the study site and by the reading center, only if quality was deemed sufficient (according to subjective judgment). BCVA, best corrected visual acuity; STARTT, Stargardt Remofuscin Treatment Trial; qAF, quantitative autofluorescence.

A total of 109 patients were screened of whom 87 patients met the inclusion criteria of STARTT.^21^ Excluded patients had a qAF value below 300 units in both eyes (9 patients), unattainable qAF imaging (3 patients), a best-corrected visual acuity (BCVA) below 0.20 in both eyes (7 patients), clinically significant abnormal blood values (3 patients), a corrected QT interval (QTc) of ≥450 ms in male subjects (1 patient), or did not proceed because of coronavirus disease 2019 (COVID-19) restrictions (1 patient). No image was acquired if it was immediately clear that a patient did not meet all inclusion and exclusion criteria. For 13 patients (25 eyes), the additional visit 1B was necessary to repeat qAF imaging. In this paper, visit 1B values were used for these subjects as the screening instead of visit 1 values. Figure 1 gives a detailed overview of the included and excluded eyes in this study. At screening, qAF imaging was performed in 202 eyes of 102 patients. At baseline (visit 2), qAF imaging was performed in 166 eyes of 84 of the included patients. There was no baseline value because of the use of wrong settings (2 patients and 3 eyes), a software failure (1 patient and 2 eyes) and because one patient refused a baseline visit.

### Quantitative Autofluorescence Imaging Acquisition

The qAF imaging was acquired using a confocal scanning laser ophthalmoscope with a 488-nm excitation light (Spectralis; Heidelberg Engineering, Heidelberg, Germany), equipped with the qAF imaging mode that consisted of the qAF reference standard (provided and calibrated by Heidelberg Engineering), and the upgrade to the HEYEX software version 5.6 or higher. Image acquisition and transfer of data to the reading center (GRADE Reading Center, Bonn, Germany) was performed according to predefined standard operational procedures.

The detailed acquisition protocol for qAF imaging required adequate pupil dilatation that was also documented by fundus reflex infrared (IR) images. Prior to qAF imaging, IR imaging followed by acquisition of standard fundus autofluorescence (FAF) was carried out. In addition to camera positioning and orientation of the image frame, particular care was taken to ensure optimal focusing (considering different chromatic aberrations between modalities). Correct detector sensitivity setting (or “gain”) was achieved. Extra care was made to ensure even illumination of the image frame, avoiding any shadowing at the image edges or corners caused by imperfect alignment of the camera. After optimizing the settings and camera alignment, light adaptation was ensured by exposing the retina to the blue excitation light (488 nm) of the qAF mode for at least 20 seconds in order to reduce photopigment absorption. Patients were instructed to keep both eyes wide open and to avoid eye movements. Operators were asked to make sure the scanning light beam remained on the center of the pupil and to compensate for any patient movement to ensure consistent exposure of blue laser light to the central macula (field 2).

Two qAF imaging sets (referred to as “movies”) for both eyes each consisting of 12 frames, covering a 30 by 30 degree field of view (768 × 768 pixels) were taken, centered on the fovea (field 2). After capturing the first movie for each eye, patients were instructed to sit back, and additionally the camera was pulled back, to reset all orientation-related settings. Followed by a rest of a few seconds, the patients were asked to return to the chin rest, subsequently the instrument position and settings were re-optimized and the second qAF movie was obtained. Before leaving the camera, operators had to check the qAF images for validation purposes. They were specifically asked to ensure non-flickering image frames, equal brightness of individual frames, and absence of any shadowing. For each movie, at least nine frames had to meet these high-standard quality criteria. If the image quality requirements were deemed not to be satisfactory, the image acquisition was repeated.

### Quantitative Autofluorescence Image Analysis

The study site and the reading center analyzed the images separately. All study site operators received a manual with a step-by-step explanation for analysis of qAF images. Readers at the reading center underwent in-person training and were provided with the grading manual. For each visit, the reading center graded both qAF movies (resulting in qAF values for movie 1 and movie 2). The study site only evaluated the qAF movie with the highest quality (according to subjective judgment). The qAF images at screening from site #5 were only analyzed by the reading center, due to understaffing during the screening period.

**Figure 2.**
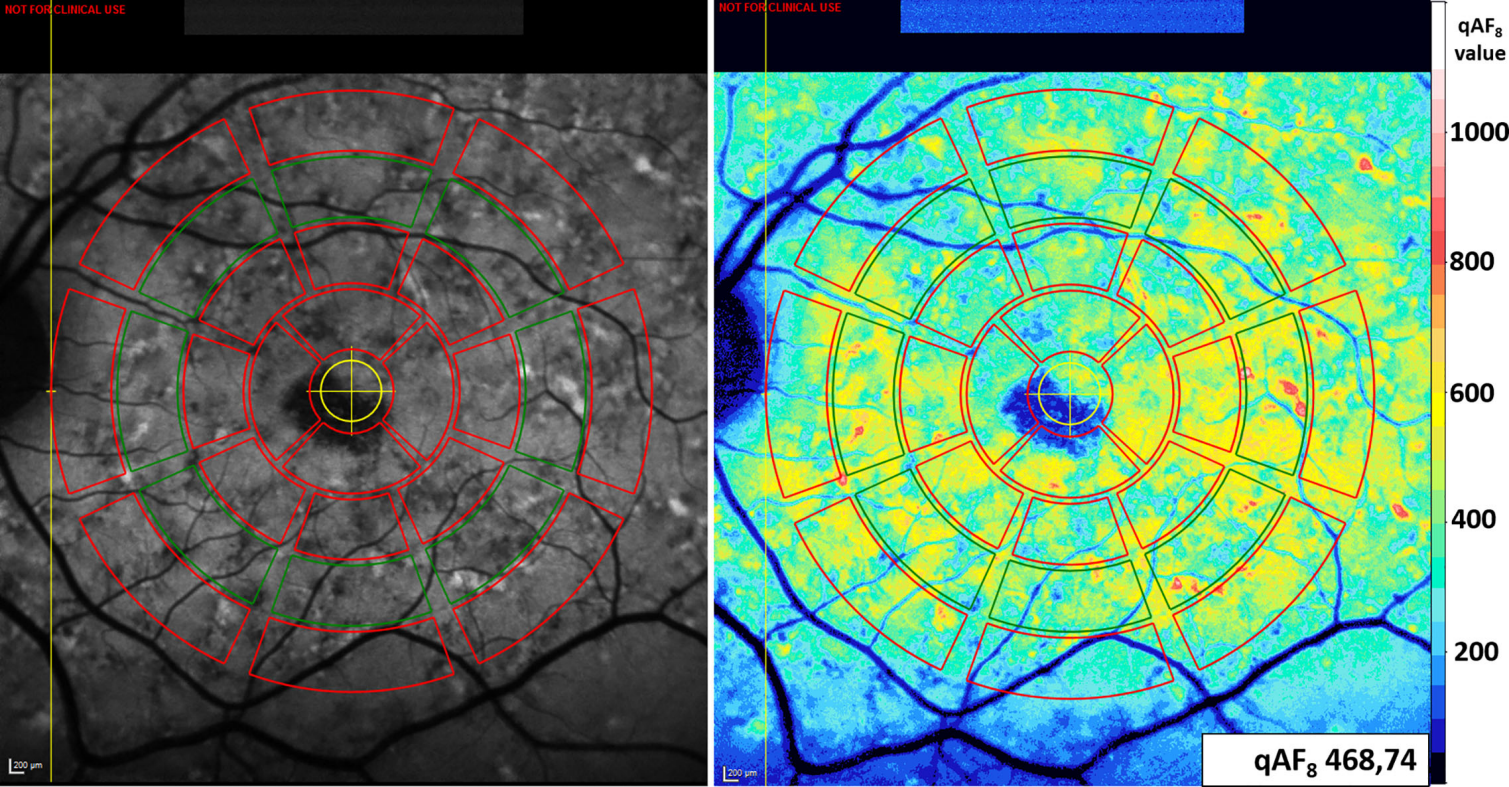
Quantitative autofluorescence (qAF) image analysis. The qAF_8_ values were determined by calculating the mean qAF values within the eight segments of the middle ring (*green* segments) of the pattern introduced by Delori et al.^18^ Please note the slight shadow inferior created by the upper eyelid leading to inaccurate low qAF levels in this image. Evaluation of qAF_8_ level was based on the whole set of images.

Images were analyzed using the qAF add-on tool in the HEYEX software (Heidelberg Engineering, Heidelberg, Germany), see Figure 2. Individual frames of the qAF movie were reviewed and only those with good quality and without artifacts were used to compute a mean color-coded non-normalized qAF image. The individual average corneal curvature measurement and patient's age were fitted to this generated qAF image. The pattern introduced by Delori et al. was placed, centered on the fovea, and its border moved in direction of the temporal border of the optic disc.^17^^,^^18^ Before analysis of the qAF level, atrophic areas and vessels were excluded by an individual segmentation based on the algorithm and with manual alterations if deemed necessary. The qAF level was calculated for each segment of the Delori pattern by comparing the mean grey level of the segment to the built-in fluorescence reference standard using the formula previously described by Delori et al.^18^^,^^22^ This formula includes the reference calibration factor, which corresponds to the used device and should compensate for using different devices in a multicenter setting. The formula also corrects for the increasing variability of lens opacities with increasing age and determines the fundus autofluorescence level relative to that which would be measured through the media of a 20-year-old emmetropic eye with average ocular dimensions. The qAF_8_ value was calculated by taking the mean of the grey levels in the eight segments of the middle ring of the Delori pattern.^17^^,^^18^

### Certification of Operators

Before the start of the clinical trial, operators from all clinical sites joined an in-person training course at the reading center. This 6-hour long course included lectures addressing the background, optimal image acquisition, and pitfalls of qAF in detail. Operators also joined practical hand-on sessions with the Spectralis device to improve their skills for achieving high quality autofluorescence (AF) images. The course also allowed interactions among operators, reading center staff, and other experts in qAF imaging. Operators who joined during the course of study were trained based on distance learning.

Prior to acquisition of images of study patients, all operators had to also gain individual certification by the reading center. Certification required successful submission of two qAF image sets, that met the predefined quality standards of qAF images covering image quality, completeness, and adherence to the acquisition protocol. Specifically, in each submitted qAF movie for certification request, at least nine frames with adequate quality had to be present, showing proper orientation and centering on the fovea, equal brightness, even illumination and absence of any shadowing of the image frames. Unfortunately, it is very difficult to prevent slight shadowing from appearing in the images. Slight shadowing of image corners is therefore allowed, as long as the image is centered on fovea. If the quality requirements were deemed not to be satisfactory, the operators received detailed advice and a resubmission of certification images was required. Only operators demonstrating capabilities to obtain qAF images meeting quality standards were certified for acquisition of qAF images during the study.

### Reproducibility Measures

Intra-visit reproducibility was calculated by comparing the qAF_8_ levels obtained from qAF movie 1 and movie 2 of the same eye and had been acquired on the same day. By comparing the screening and baseline values within the same eye, we determined the inter-visit reproducibility. Because of the adjustments between movie 1 and movie 2 of one visit, resulting in a potential longer light adaptation period before the second movie, we compared the screening value of movie 1 with the baseline value of movie 1 and the screening value of movie 2 with the baseline value of movie 2 separately. The intra- and inter-visit reproducibility were assessed using the qAF_8_ levels as graded by the reading center. To define interobserver reproducibility, we compared the grading of qAF levels as obtained by the study site to the assessment of the reading center, using the same qAF movie of the same eye that had been acquired on the same visit. Supplementary Table S1 shows an overview of the number of included and excluded eyes per reproducibility measure.

We hypothesize that operator skills will likely influence qAF repeatability. We have, therefore, compared the inter-visit reproducibility based on qAF values obtained by the same operator to results from the whole population. In addition, we have compared the inter-visit repeatability using the STARTT method (selecting the better of the two movies) to results using the individual measurements.

### Statistical Analysis

Statistical analysis was performed using the SPSS statistics package for Windows; version 25 (SPSS IBM, Armonk, NY). One-way ANOVA, Kruskal-Wallis, test and chi-square test were used to compare patient characteristics between study sites among the 102 patients who underwent qAF imaging at screening. To include the data from the two eyes, comparison of qAF levels between study sites was assessed using a linear mixed-model, in which the qAF level was the dependent variable and the study site was the main independent variable.

For all reproducibility measures, the intraclass correlation (ICC) estimate was analyzed via a single-measurements, absolute-agreement, 2-way random-effects’ model. Both the difference between two measurements (Δ*qAF*) and the mean of the qAF values ($\bar{qAF}$) was calculated to compute the repeatability using the method of Bland-Altman.^23^ A one-sample *t*-test was performed to calculate the mean bias. A *P* value of <0.05 was considered significant. The coefficient of repeatability (CR), expressed in percent, was calculated in the following way^18^^,^^23^:

$$\begin{array}{ccc} & & Coefficient\;of\;repeatability\\ & & =\;\pm 1.96\;\times \sigma \left(\frac{\Delta qAF}{\bar{qAF}}\right)\;\times \;100\% \end{array}$$

The CR corresponds to a limit of agreement containing 95% of the differences and will increase if the variability of measurements increases.

## Results

We included 102 patients with STGD1 (202 eyes) who underwent qAF imaging of which 199 eyes were gradable according to the reading center. The mean age of these patients was 36 ± 12 years, and 56 (54.9%) were women. Mean qAF_8_ value at screening as graded by the reading center was 438.04 ± 125.66 units. Table 1 shows the characteristics of the screened patients per study site. There were significant differences in qAF_8_ value (*P* < 0.001, mixed-model analysis) and disease duration (*P* = 0.003, Kruskal-Wallis test) between the sites. There were no statistically significant differences between group means of current age (*P* = 0.301, 1-way ANOVA) or age at onset (*P* = 0.785, 1-way ANOVA) between the study sites. Chi-square test showed no significant difference in sex (*P* = 0.154), however, the sites #5 and #6 had too few patients to make a valid comparison.

**Table 1.** Characteristics of Patients With Recessive Stargardt Disease 1 that Underwent Screening per Study Site

	All sites (*n* = 102)	Site #1 (*n* = 27)	Site #2 (*n* = 26)	Site #3 (*n* = 18)	Site #4 (*n* = 13)	Site #5 (*n* = 10)	Site #6 (*n* = 8)	*P* Value^a^
Female sex (%)	54.9%	55.6%	38.5%	72.2%	46.2%	80%	50%	0.154^b^
Age in years, mean ± SD (range)	36 ± 12 (18–69)	36 ± 14 (18–69)	34 ± 9 (18–59)	41 ± 13 (23–69)	40 ± 13 (24–67)	35 ± 13 (19–60)	31 ± 9 (18–44)	0.301^c^
Age at onset in years, mean ± SD	28 ± 9	27 ± 9	30 ± 8	29 ± 9	26 ± 7	31 ± 10	29 ± 9	0.785^c^
Disease duration in years, mean ± SD	8 ± 9	9 ± 10	5 ± 4	12 ± 11	15 ± 12	5 ± 4	2 ± 1	0.003^d^
qAF level in units for all available eyes at screening as measured by the reading center, mean ± SD	438.04 ± 125.66	444.64 ± 114.86	459.47 ± 133.14	371.39 ± 121.88	376.43 ± 46.90	475.20 ± 165.52	512.73 ± 80.46	< 0.001^e^

SD, standard deviation; qAF, quantitative autofluorescence.

a Comparing the six study sites.

b Chi-square test.

c One-way ANOVA.

d Kruskal-Wallis test.

e Mixed-model analysis.

**Figure 3.**
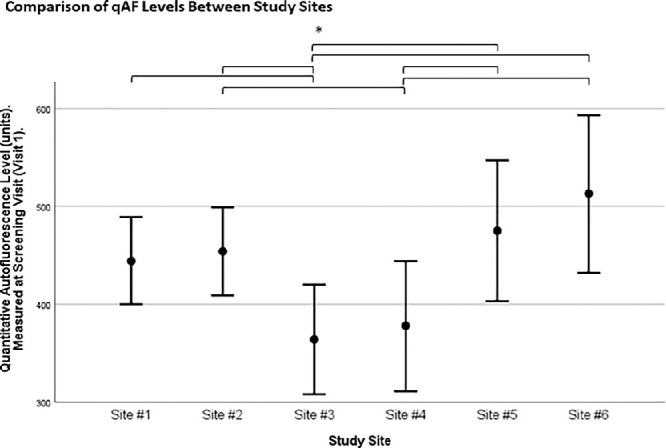
Comparison of qAF levels between study sites assessed using a linear mixed-model that included the data from two eyes of the same patient. The figure displays the model-estimated marginal means and 95% confidence interval per study site. Significant differences in qAF_8_ levels were found between several study sites. *P < 0.05.

The mixed-model showed significant differences in qAF8 values among the six study sites (*P* < 0.001). According to post hoc tests, the qAF_8_ values of sites #3 and #4 were significantly lower as compared to the other sites (*P* < 0.05), except for the comparison between sites #4 and #1. Figure 3 shows the comparison between study sites based on the estimated marginal means of a mixed-model.

Besides different qAF_8_ values among sites, there also was a statistically significant difference in disease duration as determined by Kruskal-Wallis test (*P* = 0.003). The post hoc test shows a significantly longer disease duration for site #3 as compared to sites #2 and #6, and for site #4 as compared to all other sites except #3 (*P* < 0.05, post hoc tests). Therefore, disease duration might be a possible confounder when looking at the differences in qAF_8_ value among sites. However, when we included disease duration in the mixed-model as covariate, disease duration did not play a role in determining the qAF_8_ value (*P* = 0.296, mixed-model analysis).

A total of 84 patients had a qAF8 value ≥300 units in at least one eye at screening (visit 1) and were asked for a baseline visit (visit 2). The interval between both visits was between 6 and 72 days with a mean of 20 ± 13 days.

### Reproducibility Measures

**Figure 4.**
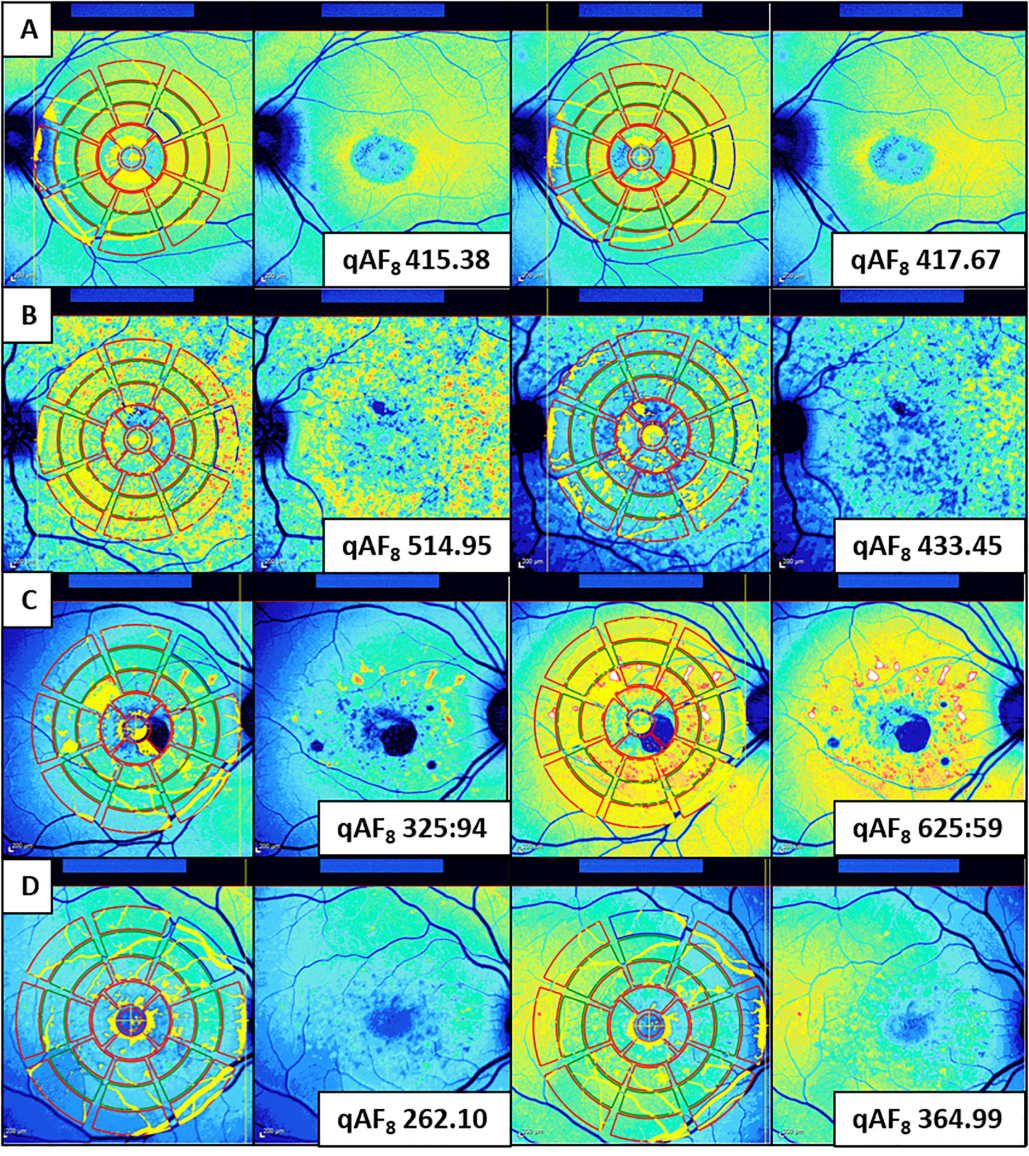
Examples of quantitative autofluorescence (qAF) repeatability. Two qAF movies are performed per visit. All orientation-related settings were reset between both movies. The reading center determined the qAF_8_ values by calculating the mean qAF values within the eight segments of the middle ring of the pattern introduced by Delori et al.^18^ In each row, the first two images belong to the first movie and the following images belong to the second movie. Row A shows an example of good intra-visit repeatability. Row B through D show examples of low intra-visit repeatability.

**Figure 5.**
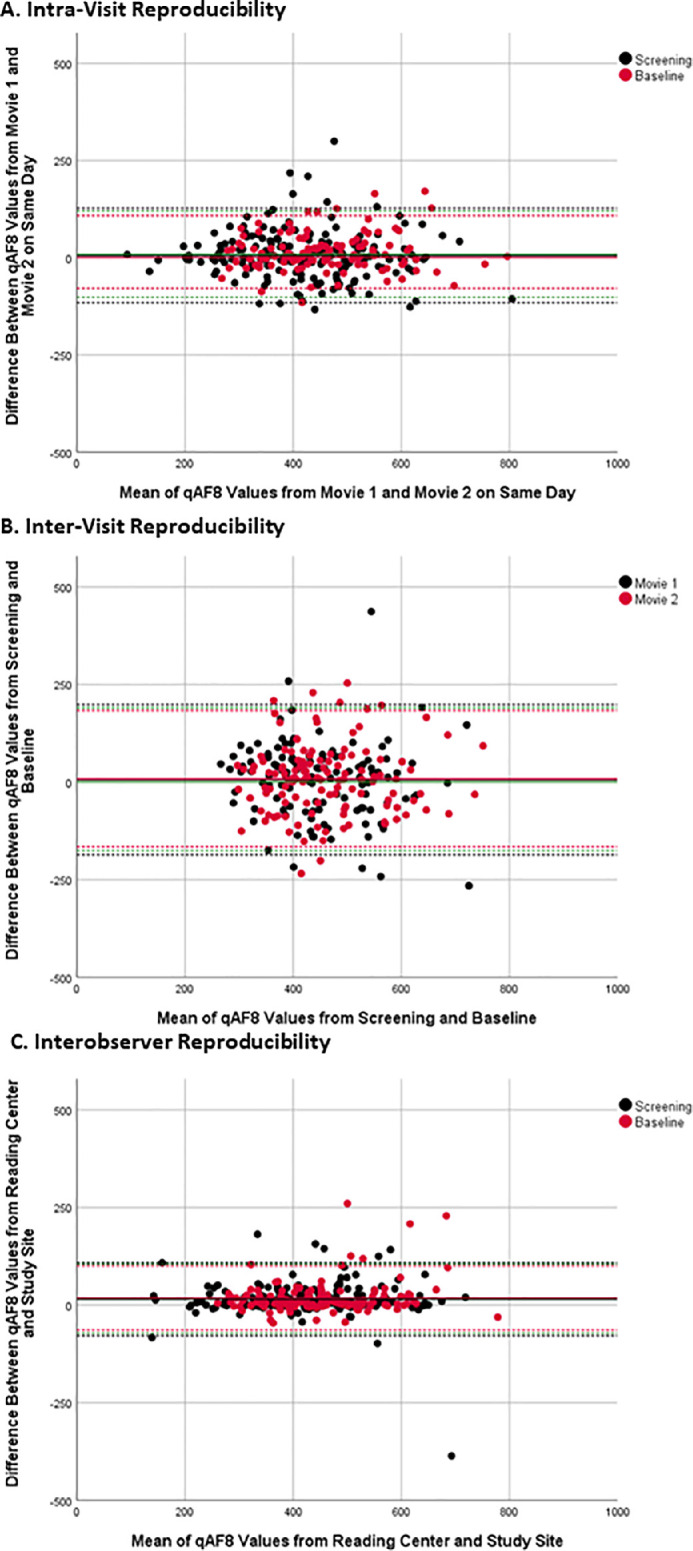
Bland–Altman plots of intra-visit reproducibility (**A**), inter-visit reproducibility (**B**), interobserver reproducibility (**C**). *Dotted lines* represent the 95% confidence interval, the *green dotted lines* represent the 95% confidence interval for all values combined.

Figure 4 shows examples of good repeatability (Fig. 4A) and low repeatability (Figs. 4B, 4C, 4D). The results for intra-visit and inter-visit reproducibility based on qAF values of the reading center are shown in Table 2. ICC showed good to excellent intra-visit repeatability for all sites, ICC of the sites ranged between 0.88 and 0.96. The variability between visits was higher with ICC of the sites ranging between 0.39 and 0.83. There was a significant average difference (*P* = 0.006, one sample *t*-test) of 9.04 ± 56.66 qAF_8_ units between both movies acquired on the same day, with the second movie having the higher value. There was no significant difference in qAF_8_ units between visits (*P* = 0.238, one sample *t*-test). Bland-Altman CR was ±26.1% for intra-visit reproducibility and ±40.5% for inter-visit reproducibility. Bland-Altman plots (Fig. 5) show the differences in qAF_8_ values for all reproducibility measures. Overall, repeatability was better (the CR was lower) for the baseline values compared to screening values and for the second movie values compared to the values of the first movie.

**Table 2.** Reproducibility of Quantitative Autofluorescence in Patients With Recessive Stargardt Disease 1 Specified per Study Site

	All Sites	Site #1	Site #2	Site #3	Site #4	Site #5	Site #6
	**Intraclass correlation, ICC estimate (95% CI)**						
Intra-visit	0.94 (0.92–0.95)	0.92 (0.88–0.95)	0.91 (0.85–0.94)	0.94 (0.89–0.97)	0.88 (0.77–0.94)	0.96 (0.92–0.98)	0.90 (0.79–0.95)
Screening values only	0.93 (0.91–0.95)	0.90 (0.83–0.94)	0.91 (0.83–0.95)	0.94 (0.88–0.97)	0.86 (0.67–0.94)	0.97 (0.94–0.99)	0.91 (0.73–0.97)
Baseline values only	0.95 (0.92–0.96)	0.96 (0.93–0.98)	0.90 (0.79–0.96)	0.93 (0.77–0.98)	0.91 (0.74–0.96)	0.84 (0.44–0.96)	0.89 (0.68–0.97)
Inter-visit	0.76 (0.69–0.81)	0.60 (0.36–0.74)	0.80 (0.66–0.88)	0.39 (−0.38–0.73)	0.77 (0.48–0.90)	0.66 (0.20–0.86)	0.83 (0.65–0.92)
Movie 1 values only	0.74 (0.63–0.82)	0.53 (0.09–0.76)	0.80 (0.57–0.90)	0.40 (–1.66–0.85)	0.69 (0.16–0.89)	0.66 (−0.07–0.90)	0.83 (0.54–0.94)
Movie 2 values only	0.78 (0.69–0.85)	0.66 (0.35–0.82)	0.80 (0.57–0.90)	0.39 (–0.88–0.79)	0.87 (0.57–0.96)	0.68 (−0.08–0.91)	0.83 (0.47–0.95)
	**One sample *t*-test of difference, mean difference in qAF units ±SD (*P* value)**						
Intra-visit	9.04 ± 56.66 (*P* = 0.006)^*^	14.86 ± 56.67 (*P* = 0.015)^*^	5.61 ± 69.94 (*P* = 0.507)	−1.25 ± 52.33 (*P* = 0.881)	11.92 ± 33.06 (*P* = 0.026)^*^	12.11 ± 61.49 (*P* = 0.281)	6.05 ± 49.78 (*P* = 0.511)
Screening values only	5.28 ± 61.89 (*P* = 0.254)	17.85 ± 68.92 (*P* = 0.070)	4.23 ± 75.80 (*P* = 0.716)	−10.11 ± 54.29 (*P* = 0.333)	9.26 ± 35.06 (*P* = 0.229)	−1.57 ± 51.04 (*P* = 0.892)	−1.98 ± 48.28 (*P* = 0.872)
Baseline values only	14.63 ± 47.55 (*P* = 0.001)^*^	10.95 ± 35.25 (*P* = 0.060)	7.9 ± 60.36 (*P* = 0.511)	19.44 ± 42.53 (*P* = 0.142)	15.00 ± 31.23 (*P* = 0.051)	36.98 ± 73.06 (*P* = 0.124)	15.24 ± 51.65 (*P* = 0.290)
Inter-visit	7.06 ± 93.33 (*P* = 0.238)	9.34 ± 108.37 (*P* = 0.455)	−14.09 ± 86.24 (*P* = 0.223)	3.16 ± 103.17 (*P* = 0.875)	20.74 ± 39.78 (*P* = 0.007)^*^	62.82 ± 110.59 (*P* = 0.012)^*^	−11.80 ± 62.00 (*P* = 0.303)
Movie 1 values only	5.74 ± 98.06 (*P* = 0.524)	19.46 ± 117.85 (*P* = 0.322)	−23.05 ± 85.51 (*P* = 0.173)	−2.52 ± 110.75 (*P* = 0.941)	23.73 ± 47.91 (*P* = 0.066)	47.33 ± 121.53 (*P* = 0.204)	−20.89 ± 61.94 (*P* = 0.197)
Movie 2 values only	8.32 ± 88.97 (*P* = 0.298)	−0.27 ± 99.14 (*P* = 0.987)	−6.03 ± 87.55 (*P* = 0.709)	7.06 ± 101.16 (*P* = 0.784)	17.55 ± 30.16 (*P* = 0.041)^*^	79.73 ± 100.30 (*P* = 0.025)^*^	−1.54 ± 62.71 (*P* = 0.928)
	**Coefficient of repeatability, CR ±%**						
Intra-visit	±26.1%	±26.2%	±33.1%	±26.3%	±18.4%	±21.4%	±20.8%
Screening values only	±29.2%	±31.7%	±37.0%	±27.4%	±19.8%	±19.8%	±18.9%
Baseline values only	±20.6%	±16.7%	±26.1 %	±21.3%	±17.0%	±22.6%	±22.5%
Inter-visit	±40.5%	±46.7%	±38.7 %	±52.5%	±20.5%	±37.3%	±25.2%
Movie 1 values only	±42.0%	±50.8%	±35.1 %	±58.1%	±24.4%	±40.1%	±26.1%
Movie 2 values only	±39.3%	±42.6%	±42.2 %	±50.2%	±15.7%	±35.3%	±24.0%

Intra-visit: movie 1 value was subtracted from movie 2 value. Inter-visit: screening value was subtracted from baseline value.

CI, confidence interval; CR, coefficient of repeatability; ICC, intraclass correlation; SD, standard deviation;

* *P* < 0.05.

The results for intergrader reproducibility are shown in Table 3. Interobserver reproducibility was excellent for almost all sites with ICC ranging between 0.87 and 0.99 and CR was ± 20.2%. Grading by the central reading center was 16.30 ± 45.09 qAF_8_ units higher compared to the grading at the study site (*P* < 0.001, one sample *t*-test). In all cases, the reading center reported higher qAF_8_ values than all study sites.

**Table 3.** Interobserver Reproducibility of Quantitative Autofluorescence in Patients With Recessive Stargardt Disease 1 Specified per Study Site

	All sites	Site #1	Site #2	Site #3	Site #4	Site #5	Site #6
	**Intraclass correlation, ICC estimate (95% CI)**						
Interobserver Reproducibility	0.95 (0.93–0.97)	0.99 (0.98–0.99)	0.98 (0.90–0.99)	0.91 (0.84–0.95)	0.87 (0.64–0.94)	n.a.^†^	0.95 (0.90–0.98)
Screening values only	0.96 (0.94–0.97)	0.98 (0.96–0.99)	0.98 (0.91–0.99)	0.90 (0.78–0.95)	0.79 (0.43–0.92)	n.a.^†^	0.90 (0.73–0.97)
Baseline values only	0.95 (0.91–0.97)	1.00 (0.99–1.00)	0.98 (0.86–0.99)	0.95 (0.88–0.98)	0.93 (0.60–0.98)	0.46 (−0.15–0.88)	1.00 (0.99–1.00)
	**One sample *t*-test of difference, mean difference in qAF units ±SD (*P* value)**						
Interobserver reproducibility	16.30 ± 45.09 (*P* < 0.001)^*^	4.22 ± 26.71 (*P* = 0.128)	21.86 ± 26.64 (*P* < 0.001)^*^	9.44 ± 68.81 (*P* = 0.357)	21.03 ± 32.28 (*P* < 0.001)^*^	n.a.^†^	9.16 ± 34.60 (*P* = 0.144)
Screening values only	14.59 ± 47.77 (*P* < 0.001)^*^	7.23 ± 33.74 (*P* = 0.129)	23.13 ± 30.22 (*P* < 0.001)^*^	8.74 ± 84.72 (*P* = 0.589)	22.64 ± 40.98 (*P* = 0.023)^*^	n.a.^†^	13.64 ± 48.54 (*P* = 0.279)
Baseline values only	18.29 ± 41.85 (*P* < 0.001)^*^	0.51 ± 13.27 (*P* = 0.804)	20.24 ± 21.55 (*P* < 0.001)^*^	10.53 ± 33.68 (*P* = 0.202)	19.42 ± 21.30 (*P* = 0.001)^*^	162.56 ± 67.53 (*P* = 0.001)^*^	4.69 ± 8.68 (*P* = 0.047)^*^
	**Coefficient of repeatability, CR ±%**						
Interobserver Reproducibility	±20.2	±20.8	±10.8	±25.4	±18.4	n.a.^†^	±15.0
Screening values only	±23.8	±26.9	±11.8	±31.3	±24.4	n.a.^†^	±22.5
Baseline values only	±18.2	±6.4	±9.0	±18.8	±10.6	±24.3	±3.1

Interobserver reproducibility: site value was subtracted from reading center value.

CI, confidence interval; CR, coefficient of repeatability; ICC, intraclass correlation; SD, standard deviation;

† Not applicable due to protocol change for this site.

* *P* < 0.05.

Unfortunately, it is very difficult to prevent slight shadowing from appearing in the images. Note that these shadows, mostly created by the upper eyelid or pupil border, may lead to inaccurate low qAF level. For this figure, we picked one representative qAF image, however, the evaluation was based on the whole set of at least nine images.

Table 4 shows that repeatability will change depending on the included values. We have compared the results from the whole population to inter-visit reproducibility based on qAF values obtained by the same operator and the values based on STARTT approach (using the highest value of the two movies acquired on the same day). Using this approach, ICC increased to 0.82 (0.74–0.87) when all the data are used, compared with 0.78 (0.70–0.84) if only data taken by the same operators are used and 0.76 (0.69–0.81) when individual measurements of the whole population are used. Notably, if we include only same operators, there is a significant mean difference in qAF units (*P* = 0.047, one sample *t*-test). CR remains 40.5% if operators stay the same, however, it decreases to 34.6% when the STARTT method is being used.

**Table 4.** Inter-Visit Reproducibility Depending on Design

	Individual Measurements of Whole Population (*n* = 244)	Individual Measurements Always Same Operators (*n* = 151)	Method STARTT (Using The Best of Two Movies) (*n* = 134)
	**Intraclass correlation, ICC estimate (95% CI)**		
Inter-visit	0.76 (0.69–0.81)	0.78 (0.70–0.84)	0.82 (0.74–0.87)
	**One sample *t*-test of difference, mean difference in qAF units ±SD (*P* value)**		
Inter-visit	7.06 ± 93.33	15.57 ± 95.51	−5.79 ± 83.90
	(*P* = 0.238)	(*P* = 0.047)^*^	(*P* = 0.426)
	**Coefficient of repeatability, CR ± %**		
Inter-visit	±40.5%	±40.5	±34.6

Inter-visit: screening value was subtracted from baseline value.

CI, confidence interval; CR, coefficient of repeatability; ICC, intraclass correlation; SD, standard deviation.

* *P* < 0.05.

## Discussion

Increased fundus AF intensity, a signal originating from RPE lipofuscin, is considered a hallmark of STGD1 and, therefore, qualifies as an outcome measure for interventional clinical trials aiming to improve the visual outcome by lowering the lipofuscin accumulation and its toxic effects in patients.^15^^–^^17^ Currently, most trials focus on the objective measurement of the extent of RPE atrophy on short-wavelength fundus autofluorescence (SW-AF) imaging.^20^ However, because of the relative slow and variable progression rate of RPE atrophy as well as sometimes not well-defined borders, the sensitivity to detect a short-term treatment effect is low*.*^24^^–^^28^ Besides, measurement of RPE atrophy can only be used when cell death is already present and can no longer be prevented.

Even with strict, standardized imaging protocols, there is an inherent variability in routine AF imaging. Recent adaptations now allow for quantification of the AF signal by means of qAF.^18^^,^^19^ This could improve the reproducibility in individual patients over time and would facilitate the use of AF intensity across multiple sites in clinical trials. As qAF levels are elevated already early in the STGD1 course,^17^^–^^19^ qAF has the potential to become a valid and sensitive surrogate end point for clinical trials aiming to treat STGD1. It is still critical that skilled operators use the utmost care during image acquisition. In practice, proper centering and the prevention of artifacts from eyelids and eye movement remains challenging.^12^ Poor image quality will lead to inaccurate qAF values. Specifically poor image quality usually manifests as a low qAF value. In a placebo-controlled clinical study, artificially low values may influence the difference in treatment effect of the active compared to the placebo arm. Working toward validation of qAF for clinical studies, this paper evaluates the repeatability of qAF in a multicenter clinical trial setting.

To our knowledge, this paper is the first to review qAF data in a large sample of STGD1 subjects on multiple visits and in a multicenter clinical study set up in which different devices were used by several operators, involving several graders and an independent reading center. The data used for this paper were obtained from subjects who agreed to participate in the STARTT. Because the mechanism of action of remofuscin tested with STARTT is to facilitate the elimination of lipofuscin from the RPE over time, qAF was seen as a direct method of assessing its action. Other anatomic and functional end points will also be evaluated in due course. It is important to note that STARTT was not designed or intended to validate the use of qAF. The conclusions from this paper should be interpreted in this context. However, the protocol required two qAF movies on two occasions (four sets of images) before any test product was used thereby providing a unique dataset for evaluating qAF repeatability.

The qAF8 repeatability in our dataset was ±26.1% for intra-visit, ±40.5% for inter-visit, and ±20.2% for the interobserver reproducibility measures (Bland-Altman CR). Single center studies in eyes without retinal pathology presented Bland-Altman CRs of 6% to 12% for intra-visit repeatability and 7% to 14% for inter-visit repeatability (interval up to 64 days).^18^^,^^29^^,^^30^ Studies that included patients with retinal pathology presented an intra-visit repeatability of ±10.3% in patients STGD1,^17^ ±8.8% in patients with bull's eye maculopathy (in some patients caused by mutations in *ABCA4*),^31^ ±9.3% in patients with a retinal dystrophy caused by *ABCA4* or *PRPH2* mutations,^32^ ±7% in patients with Best vitelliform macular dystrophy,^33^ and ±8.2% in patients with age-related macular degeneration (AMD).^34^ So far, the inter-visit repeatability has only been examined in patients with AMD. The agreement after 3 and 6 months follow-up was ±8.3% and ±9.8% in eyes without retinal changes and ±18.3% and ±20.2% in eyes with changes in drusen volume. As expected from a multicenter study, compared to these previously reported numbers from single center studies, our study shows higher repeatability coefficients.

The intra-visit measurements, performed by the same operator on the same day, were found to be the most consistent. The ICC was good to excellent across all sites at 0.94 (0.92–0.95). This result is in line with those of Reiter et al., who conducted the only other study that used ICC to estimate the reliability of qAF.^34^ They showed excellent ICC for qAF images of patients with AMD on the same day (ICC = 0.98) and after 3 to 6 months follow-up (ICC = 0.97 and 0.98, respectively). Together with a mean difference of 9.04 ± 56.66 units and a CR of ±26.1%, our data suggest a reasonable intra-visit consistency. Yet, the qAF_8_ values of the second movie were significantly higher as compared to the first movie of the same day (*P* = 0.006, one sample *t*-test).

Both Delori et al. and Greenberg et al. have reported second movie values to be higher in comparison to the first movie and related this to a systematic error in the zero level.^18^^,^^29^ However, the second movie might have benefitted from additional light exposure during first movie acquisition. Insufficient light adaptation leaves room for photopigment absorption, and is therefore considered a source of error leading to lower values.^35^ Moreover, the degree of pupil dilatation as well as patient motivation and instructions given to patients need to be considered as well. Equally important is the fact that the color-coded qAF view is not available to the operator at the time of image acquisition. Therefore, greatest attention must be given to ensure even illumination based on grey level intensities in the live image throughout the whole acquisition process. With our current experience, even the best photographer cannot always prevent slight shadowing from appearing in a qAF image. Unfortunately, slight shadowing may lead to inaccurate low qAF levels of that image. To minimize this confounder, evaluation of qAF_8_ levels was based on a set of at least nine qAF images in the current study. Besides, the sensitivity setting (to avoid overexposure) for patients with STGD1 would also be different for a STGD1 cohort versus healthy eyes. Technical improvements of the qAF software are needed to aid the operator, like a direct view of the color-coded image and software driven feedback which shows the overexposure and uniformity of the image.

The results for the inter-visit comparisons were less consistent between sites as compared to those for intra-visit reproducibility. However, differences across sites need to be interpreted with caution because some sites had a relatively small sample size (range = 6–27 subjects enrolled). ICC varied among sites and ranged from poor (0.39) to good (0.83). The best ICC for the inter-visit images were obtained by those sites with the most experienced operator(s) (e.g. site #6, 0.83 [0.65–0.92]). The sites with good ICC values also showed small inter-visit mean differences and lower CR. However, ranking by the three comparative indices failed to identify a single site that performed clearly and consistently better than the others. Differences across sites may, to a certain extent, reflect differences in the subject population enrolled due to referral patterns or duration of disease. At screening, we noticed significant differences in qAF_8_ values among the six study sites. The patients in the sites with lower qAF_8_ levels also showed a significantly longer disease duration. A longer disease duration might cause the reduction of AF signal levels because qAF decreases in advanced disease because of increased lesion size and the formation of dark flecks.^16^ These patients are generally older. However, when including disease duration and/or age in the mixed-model as covariate, these did not unduly affect the qAF_8_ value. The differences in qAF_8_ levels among sites might, therefore, be better explained by a difference in genetic mutations or other as yet unknown characteristics of patients. Patients with severe variants in the *ABCA4* gene reach qAF_8_ ceiling levels faster.^16^^,^^17^ In addition, qAF_8_ values vary with ethnicity,^29^ although 94% of the total cohort in the STARTT study described themselves as White.^21^ Unfortunately, the current study was not designed to be able to perform a comprehensive analysis between patient characteristics and qAF levels, and further investigation is required.

Compared to intra-visit results, inter-visit results are less strong with ICC at 0.76 (0.69-0.81), mean difference of 7.06 ± 93.33 units (*P* = 0.238), and CR of ±40.5%. The reasons for the reduction in consistency across visit dates are not completely understood. It is assumed that the true RPE lipofuscin levels would not have drastically changed in the space of a few weeks without treatment. Although reasonable, given the slow progression of STGD1, it is also possible that a change in qAF reflects the additional formation of either lipofuscin or the development of dark flecks and/or atrophy.^15^^–^^17^ A more likely explanation for the inter-visit variability is the involvement of more than one operator at different visits. If the results from sets obtained by the same operator were to be compared to results from the whole population, differences are noted. When the inter-visit images were taken by the same operators, qAF_8_ values of the baseline visit (visit 2) were significantly higher as compared to the values at screening (visit 1, *P* = 0.047, one sample *t*-test). Assuming that higher values indicate better qAF images, it seems that individual operators experience a learning curve. This emphasizes the importance of thorough operator training before the start of a clinical trial.

Within the current design, image analysis did not necessarily involve the operator who acquired the image. However, to improve image quality, operators should both acquire and analyze the qAF images as the feed-back from image analysis will help to improve qAF image acquisition. Both qAF image acquisition and image analysis are operator-dependent. The use of multiple operators and different persons who analyze the images is inevitable for a multicenter clinical trial. A central reading center is recommended to perform the final grading of images in an objective and standardized way, reducing the human component of image analysis. In our study, the coefficient of repeatability between the reading center and the qAF value as determined by the operator was ±20.2%. Differences between reading center and sites are likely based on the selection of the frames included in the averaged image and the manual adjustment of the atrophy- and vessel exclusion algorithm. These procedures should be further standardized to improve interobserver agreement.

Although qAF reproducibility values seem reasonable, the size of the standard deviation may prove an issue with future sample size calculations. When using qAF as a clinical trial end point, increased variability may have to be considered. Within the STARTT, the reading center will use the higher value of two movies to evaluate the efficacy of remofuscin. This approach appears to be justified. When this method is used, inter-visit ICC increases to 0.82 (0.74–0.87), the mean difference becomes -5.79 ± 83.90 (*P* = 0.426), and the CR improves to ±34.6%.

In conclusion, our qAF repeatability results are the first from a prospective multicenter collection of data. Previous publications included data obtained from a single center with very few operators acquiring images. Our data indicate that the variability in qAF levels is more strongly associated with image acquisition rather than analysis. Well-trained and experienced operators following the same protocol and procedures are crucial when using qAF as an end point in multicenter clinical trials. The engagement of an experienced reading center with rigorous quality assessments adds confidence to the results. However, variability cannot be eliminated altogether. In an ideal world, fewer operators should provide image sets, but this is often impractical in a multicenter study with a long follow-up period. Despite this, qAF could be a useful method for assessing changes in autofluorescence from RPE. Longitudinal studies with a longer review period are necessary to develop a full picture of the reliability of qAF as an outcome measure.

## Supplementary Material

Supplement 1
