# Supplementary material for: Repeatability of Quantitative Autofluorescence Imaging in a Multicenter Study Involving Patients With Recessive Stargardt Disease 1
Source: Transl Vis Sci Technol. 2023 Feb 1;12(2):1. doi: 10.1167/tvst.12.2.1 (PMC9904328; doi:10.1167/tvst.12.2.1)
Supplement: Supplement 1 [file tvst-12-2-1_s001.pdf]

Supplemental Table 1. Overview of the number of included and excluded eyes per reproducibility measure

	Included Values	Number of Included Eyes	Excluded Values
Intra-Visit Reproducibility	Screening Values	180/202 eyes, 89.1%	Insufficient quality of:<br>- Measurement 1 (4 eyes)<br>- Measurement 2 (2 eyes)<br>- Both measurements (16eyes)
	Baseline Values	121/166 eyes, 72.9%	Not graded by reading center due to incomplete data (8 eyes)<br>Insufficient quality of:<br>- Measurement 1 (15 eyes)<br>- Measurement 2 (9 eyes)<br>- Both measurements (13 eyes)
Inter-Visit Reproducibility	Movie 1 Values Only	119 / 166 eyes, 71.7%	Insufficient quality of:<br>-Screening value (11 eyes)<br>-Baseline value (31 eyes)<br>-Both screening and baseline value (5 eyes)
	Movie 2 Values Only	125 / 166 eyes, 75.3%	Insufficient quality of:<br>-Screening value (11 eyes)<br>-Baseline value (26 eyes)<br>-Both screening and baseline value (4 eyes)
Interobserver Reproducibility	Screening Values	163/202 eyes, 85.6%	Insufficient quality according to:<br>-Study site (3 eyes)<br>-Reading center (16 eyes)<br>-Not graded by site #5 (20 eyes)
	Baseline Values	140/166 eyes, 84.3%	Not graded by reading center due to incomplete data (8 eyes)<br>Insufficient quality according to:<br>-Study site (5 eyes)<br>-Reading center (21 eyes)
